# The Involvement and Manifestations of SARS-CoV-2 Virus in Cardiovascular Pathology

**DOI:** 10.3390/medicina61050773

**Published:** 2025-04-22

**Authors:** Sofia Teodora Hărșan, Anca Ileana Sin

**Affiliations:** 1 Department of Pathophysiology, George Emil Palade University of Medicine, Pharmacy, Science, and Technology of Târgu Mureș, 540139 Târgu Mureș, Romania; 2 Doctoral School of Medicine and Pharmacy, George Emil Palade University of Medicine, Pharmacy, Science, and Technology of Târgu Mureș, 540139 Târgu Mureș, Romania; 3 Department of Cellular and Molecular Biology, George Emil Palade University of Medicine, Pharmacy, Science, and Technology of Târgu Mureș, 540139 Târgu Mureș, Romania

**Keywords:** SARS-CoV-2, COVID-19, virus, cardiovascular, post-COVID-19 condition

## Abstract

Although the acute phase of the COVID-19 pandemic has subsided, the emergence of the post-COVID-19 condition presents a new and complex public health challenge, characterized by persistent, multisystem symptoms that can endure for weeks or months after the initial infection with the SARS-CoV-2 virus, significantly affecting survivors’ quality of life. Among the most concerning sequelae are cardiovascular complications, which encompass a broad spectrum of conditions, including arrhythmias, myocardial damage, or postural orthostatic tachycardia syndrome. This narrative review explores the burden of the SARS-CoV-2 infection on cardiovascular health by reviewing the latest and most relevant findings in the literature and highlighting different aspects of COVID-19’s cardiovascular involvement. This review investigates the pathophysiological mechanisms underlying cardiovascular involvement in the post-COVID-19 condition, with a focus on direct viral invasion via ACE2 receptors, immune-mediated cardiovascular injury, cytokine storm, systemic inflammation, endothelial dysfunction, and mitochondrial injury. The interplay between pre-existing cardiovascular diseases, such as hypertension, atherosclerosis, diabetes, and atrial fibrillation, and COVID-19 is also explored, revealing that individuals with such conditions are at heightened risk for both severe acute illness and long-term complications. Long-term immune activation and the persistence of viral antigens are increasingly recognized as contributors to ongoing cardiovascular damage, even in individuals with mild or asymptomatic initial infections. As the healthcare system continues to adapt to the long-term consequences of the SARS-CoV-2 pandemic, a deeper understanding of these cardiovascular manifestations is essential. This knowledge will inform the development of targeted strategies for prevention, clinical management, and rehabilitation of affected patients. Furthermore, the insights gained from the intersection of COVID-19 and cardiovascular health will be instrumental in shaping responses to future viral epidemics, highlighting the necessity for multidisciplinary approaches to patient care and public health preparedness.

## 1. Introduction

The COVID-19 pandemic has significantly impacted global health, economies, and the daily lives of everyone since its emergence in early 2020. Caused by the SARS-CoV-2 coronavirus, COVID-19 disease presents a wide range of symptoms, according to the information provided by the World Health Organization, from mild cases involving fever, cough, fatigue, and loss of taste and smell to serious symptoms like difficulty breathing, dyspnea, confusion, or chest pain that can be life-threatening [1]. As of April 2025, the World Health Organization has reported over 777 million confirmed COVID-19 cases globally [1].

Although humanity has passed the critical period of the pandemic caused by the SARS-CoV-2 virus, which first appeared in Wuhan Province, China, a new epidemic has emerged: post-COVID-19 syndrome. The World Health Organization defines this syndrome as the persistence of COVID-19 symptoms or the development of new symptoms following the remission of an acute infectious episode of the SARS-CoV-2 virus, affecting at least one organ system 3 months after the initial infection and which cannot be explained by another diagnosis or disease [1].

A study of the Italian population, published since the beginning of the pandemic, in 2020, found that over 80% of patients who had experienced an infection with the SARS-CoV-2 virus complained of the persistence of at least one symptom after the resolution of the acute episode of the disease [2].

A broad spectrum of more than two hundred symptoms is considered to be part of the long-COVID syndrome, and the Centers for Disease Control and Prevention in the United States list the following as the most common: difficulty concentrating and memory problems, persistent fatigue, breathing problems, persistent cough, sleep disorders, bowel problems, rapid heartbeat, difficulty perceiving taste and smell, and recurrent migraines [3].

Cardiovascular symptoms include persistent chest pain, shortness of breath, and fatigue, both with exertion and at rest, as well as autonomic manifestations, such as postural orthostatic tachycardia, and the most diagnosed pathological entities were arrhythmias and heart rhythm disorders, myocardial infarction, and myocarditis [4]. These symptoms affect the quality of life of patients and represent a threat to global public health. It is assumed that infection with the SARS-CoV-2 virus will modify the long-term trajectory of many chronic heart diseases and change the approach to viral epidemics.

The disease caused by the SARS-CoV-2 virus can have significant comorbid interactions with other infectious diseases such as tuberculosis and HIV/AIDS. These co-infections are especially concerning because all three diseases target the lungs, weaken the immune system, and can trigger cytokine storms, which cause severe inflammation and tissue damage. A study published by Udoakang et al. talks about high-burden areas, like Africa, where HIV and tuberculosis are already major public health issues and where COVID-19 has strained healthcare systems and reversed progress in disease control [5]. Patients diagnosed with HIV/AIDS or tuberculosis are more susceptible to severe COVID-19 outcomes—such as prolonged illness, higher hospitalization rates, and increased mortality—due to pre-existing immunosuppression and lung damage, with COVID-19 also capable of reactivating latent TB and potentially leading to long-COVID complications that further hinder recovery [5]. Another study on the African population by Kazmi et al. highlights the worsening effect of the pandemic on the burden of viral hepatitis in this area, where over 100 million individuals are infected with hepatitis virus B or C [6]. By compounding the challenges of a fragile healthcare infrastructure and exacerbating existing comorbidities, patients with chronic liver disease are at heightened risk for severe COVID-19 outcomes due to immune-mediated liver injury, hypoxia, and drug-induced hepatotoxicity, while co-infection with HIV/AIDS further accelerates progression to cirrhosis and liver cancer, which is particularly troubling given the widespread disruption of hepatitis diagnostics, treatment access, and vaccination programs amid the pandemic [6].

In this narrative review, we discuss the long-term cardiovascular problems that COVID-19 causes, the pathophysiology of long-term cardiovascular complications, and the role of cardiovascular pathologies that pre-exist acute infection and their involvement in the long-COVID syndrome.

## 2. Materials and Methods

A comprehensive literature search was conducted using PubMed and Google Scholar databases to identify studies related to cardiovascular pathology in the context of COVID-19 (SARS-CoV-2 infection). The search was carried out up to March 2025, using the following keywords and their combinations: “COVID-19”, “SARS-CoV-2”, “Cardiovascular”, “Myocarditis”, “Thrombosis”, “Endothelial dysfunction”, “Cardiac injury”, “post COVID-19 syndrome”, “post COVID-19 condition” and “long-COVID”. Boolean operators (AND, OR) were applied to refine the search.

Inclusion Criteria:

- Articles published between January 2020 and March 2025;
- Studies published in English;
- Original research, systematic reviews, meta-analyses, and narrative reviews focused on cardiovascular manifestations or complications related to COVID-19.

Exclusion Criteria:

- Articles published prior to 2020 to avoid outdated pre-pandemic data;
- Studies not directly addressing cardiovascular involvement in COVID-19.
- Non-peer-reviewed articles, conference abstracts, letters to the editor, and case reports with insufficient data.

**Figure 1:**
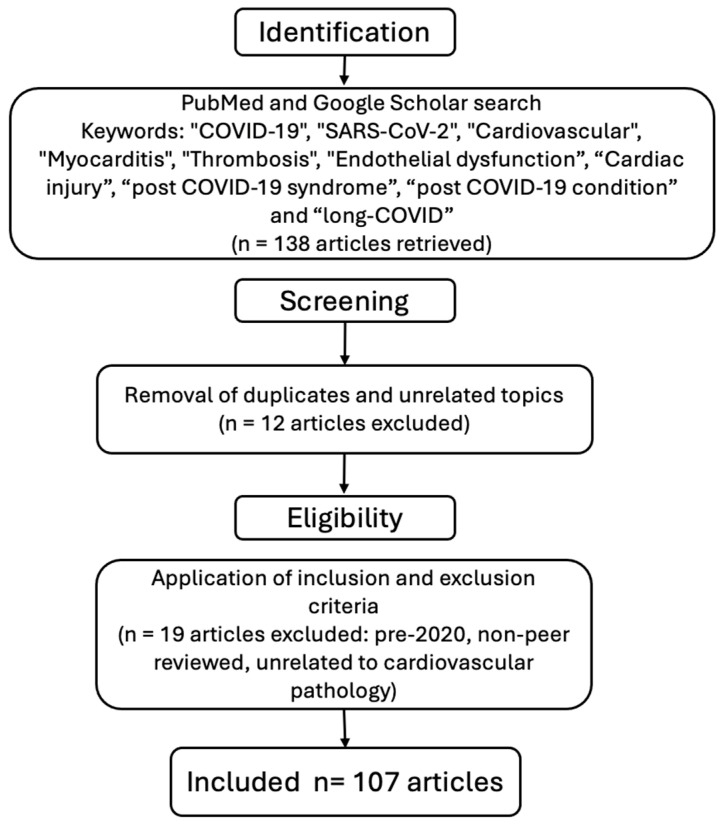
Flowchart illustrating the methodology applied to article selection in this narrative review.

Although this study adopts a narrative review approach, priority was given to high-quality peer-reviewed articles indexed in PubMed and Google Scholar to ensure scientific reliability. Formal quality assessment tools such as the PRISMA checklist were not applied due to the narrative nature of this review; however, careful selection criteria were implemented to maintain relevance and accuracy. The study selection process is summarized in Figure 1. This flowchart outlines the steps followed in the literature search, screening, and selection of articles included in this narrative review based on the predefined inclusion and exclusion criteria.

This narrative review is subject to several limitations that should be acknowledged. First, as a narrative rather than a systematic review, it lacks a standardized methodology for study selection, which may introduce selection bias. The included studies exhibit considerable heterogeneity in terms of design, population demographics, geographic settings, and definitions of both the post-COVID-19 condition and cardiovascular outcomes, limiting the generalizability of our conclusions. Additionally, the evolving definition of the post-COVID-19 condition presents challenges, as many studies cited were conducted prior to the establishment of standardized diagnostic criteria, leading to variability in symptom duration and classification. Most available data focus on short- to medium-term outcomes, with long-term cardiovascular consequences still under investigation. Furthermore, confounding factors such as coexisting conditions, particularly diabetes, hypertension, and obesity, complicate the ability to attribute cardiovascular effects solely to SARS-CoV-2 infection.

## 3. Pathogenesis of the SARS-CoV-2 Virus

Coronaviruses are microorganisms with a crown-like surface envelope, an appearance given by the presence of surface spike proteins, with genetic material containing single-stranded RNA, and which are part of the subfamily Coronaviridae Orthocoronavirinae. The SARS-CoV-2 virus, a species of coronavirus discovered in 2020 in the Wuhan province of China, is responsible for the illness of over seven hundred million people and over seven million deaths globally by 13 April 2024 [7]. This microorganism is one of three coronaviruses of animal origin that have become pathogenic for the human species, along with SARS-CoV and MERS-CoV, which have each raised global health problems [8].

The SARS-CoV-2 virus infects the host organism through the respiratory tract by binding to the receptor domain on a surface protein, highlighting the cytokine storm theory. The entry of this virus into the host organism is mediated at the cellular level by the spike glycoprotein (S), which comprises two subunits: S1 and S2 [9]. The S1 subunit is the one that carries out the entry of the virus into the host cells through the direct interaction between the angiotensin-converting enzyme 2 (ACE2) and the receptor-binding domain. By binding to ACE2 receptors, which are highly expressed not only in respiratory epithelial cells but also in vascular endothelial cells and cardiomyocytes, an immune-mediated injury is started. The virus’s entry into host cells triggers an imbalanced immune response characterized by a weak interferon reaction but an overactivation of pathogenic Th1 cells and intermediate monocytes. These immune cells release proinflammatory cytokines, including granulocyte–macrophage colony-stimulating factor (GM-CSF) and interleukin-6 (IL-6), which further amplify the inflammatory cascade by stimulating monocytes to produce excessive amounts of IL-6, tumor necrosis factor-alpha (TNF-α), and other cytokines [10].

The cytokine storm is exacerbated by the downregulation of ACE2 due to viral binding, leading to an accumulation of angiotensin II (AngII). This peptide promotes vasoconstriction, oxidative stress, and proinflammatory signaling through the angiotensin type 1 receptor (AT1R), activating nuclear factor–kappa B (NF-κB) and disintegrin and metalloproteinase 17 (ADAM17). ADAM17, in turn, cleaves the membrane-bound IL-6 receptor (mIL-6R) into its soluble form (sIL-6R), facilitating IL-6 trans-signaling via glycoprotein 130 (gp130) and signal transducers and activators of transcription 3 (STAT3). This pathway drives further cytokine production, including monocyte chemoattractant protein-1 (MCP-1) and IL-8, which recruit additional immune cells and perpetuate endothelial dysfunction [11].

Early in the infection, the respiratory tract exhibits the highest viral load, making it the primary site for viral replication and transmission. Consequently, early antiviral therapy targeting lung tissue can reduce viral replication and the risk of transmission. Aerosolized treatments, such as inhaled nano-emulsions (NEs) carrying antiviral drugs like remdesivir, show promise due to their rapid onset, targeted lung delivery, and reduced systemic side effects. Notably, the incorporation of ACE2-binding peptides in these formulations enhances the binding efficiency to lung epithelial cells, improving drug retention and targeting [12]. Moreover, the evolving viral variants, including mutations that affect the spike protein, require adaptive treatment strategies, and NEs have demonstrated good efficacy against both wild-type and mutant strains of SARS-CoV-2. Additionally, the upregulation of ACE2 in lung tissue during acute pneumonia could potentially accelerate viral transmission, suggesting that combination therapies involving antiviral and anti-inflammatory drugs could provide a more comprehensive treatment approach for COVID-19 and related respiratory diseases [13].

A study published in 2024 in the *Journal of Clinical Investigation* highlights the evolving understanding of viral variants and vaccine efficacy [14]. It highlights how newer SARS-CoV-2 variants may present challenges to immune responses, with some variants able to evade immunity induced by previous infections or vaccines. In addition, the study explores the promising role of human monoclonal antibodies targeting unusual sites on the spike protein, which may provide more robust protection against these variants. As viral evolution continues, adaptive vaccination strategies and monoclonal antibody treatments remain crucial in managing the progression of the pandemic [14].

An article published by Hiroyuki Yoshitomi discusses how the immune response in COVID-19 patients is influenced by T cells, specifically peripheral helper T cells. These cells play a role in controlling inflammation and the adaptive immune response. The research highlights the importance of understanding T-cell responses for developing better therapeutic strategies. It also underscores the significance of peripheral helper T cells in modulating the immune response to SARS-CoV-2, which could help improve disease management and vaccine design [15].

A study published in 2024 highlights that vaccination, especially with three doses, significantly improved immune responses and reduced the severity of COVID-19 in elderly patients. It also shows that different variants of the virus trigger different humoral responses, and vaccination generally provides better protection against severe outcomes, especially in the elderly. Th2 cells, linked to lung protection, were elevated in vaccinated individuals, emphasizing the vaccines’ role in preventing immune-driven lung damage [16].

## 4. Pathophysiology of Cardiovascular Involvement

The COVID-19 cytokine storm drives cardiovascular complications via inflammatory cascades. The excessive release of cytokines, particularly IL-6 and TNF-α, directly damages the vascular endothelium, leading to increased permeability, microthrombosis, and coagulopathy [11]. Neutrophil extracellular traps (NETs) further contribute to thromboinflammation by promoting clot formation and endothelial injury. In the heart, the cytokine storm induces myocardial inflammation (myocarditis), destabilizes electrophysiology (increasing arrhythmia risk), and impairs contractility, potentially leading to acute heart failure. Additionally, systemic inflammation and hypercoagulability elevate the risk of myocardial infarction, stroke, and venous thromboembolism [9].

The susceptibility of the cardiovascular system to COVID-19 infection is well known now and requires close monitoring of patients with pre-existing heart disease. Within the immune-mediated cardiovascular injury model, when the SARS-CoV-2 virus reaches cardiac muscle cells through the angiotensin-converting enzyme 2 (ACE2) receptor, it enters the myocyte and promotes a direct cytotoxic effect and the attraction of immune cells such as lymphocytes, neutrophils and monocytes, natural killer (NK) cells, and T and B lymphocytes [17]. These cells are attracted through chemotactic action by the major regulator of monocytes, MCP-1, and once monocytes differentiate into activated macrophages, the release of the following proinflammatory cytokines occurs: interleukin-1b (IL-1b), interleukins 21, 17, and 22, and tumor necrosis factor-alpha (TNF-a). Recent findings in ACE2 biology reveal the presence of a truncated subtype, ACE2δ (or ACE2-107), which is upregulated by interferon but does not bind the SARS-CoV-2 spike protein, suggesting it is unlikely to increase host susceptibility to infection. Additionally, the intracellular domain of ACE2, released upon cleavage, may play a role in gene regulation similar to activation-induced cell death (AICD) pathways, though its exact function remains unclear [18]. These insights reflect the complex and still evolving understanding of ACE2’s role in COVID-19 pathogenesis and immune regulation.

The SARS-CoV-2 virus directly induces myocardial cell damage, primarily through the cytokine storm that affects lung tissue and induces hypoxia and blood vessel damage [15]. Thus, it increases the pressure in the pulmonary circulation and implicitly preloads, leading to increased right ventricular pressure. This pressure increase leads to cardiac stress that can culminate in rhythm disorders (heart blocks, atrial fibrillation, and ventricular disorders), heart failure (compensated or decompensated), acute coronary syndrome, and even cardiogenic shock [19].

COVID-19 disease causes dysfunction of the blood vessel endothelium, either by a direct mechanism, direct infection of endothelial cells (due to the ACE2 receptor), or an indirect mechanism, by promoting a proinflammatory state and primary inflammation of blood vessels and through a cytokine storm [20]. Microvascular injury can expose the endothelium to the subendothelium and collagen, thereby activating platelets and tissue factor (TF) in the subendothelium, which, in turn, activates coagulation factor seven, FVII to FVIIa. In COVID-19, microvascular injury is a hallmark of severe disease, driven by the overexpression of tissue factor (TF) in lung epithelial cells and immune cells. This initiates the coagulation cascade and promotes thrombosis. Circulating extracellular vesicles rich in this factor further amplify clotting and inflammation. These findings suggest that tissue factor plays a central role in COVID-19-associated coagulopathy and could be targeted therapeutically to prevent thrombotic complications [21].

Thrombotic events in SARS-CoV-2 infection are linked to blood flow stasis, endothelial dysfunction, and platelet activation. These events are more common in severe cases and may persist after recovery. Inflammatory responses and interactions with ACE2 or toll-like receptors may trigger clotting, while anticoagulant and antiplatelet therapies show benefits to patients [22]. In response to inflammation, neutrophil degranulation occurs, complement is activated with the C3a, C5a, and C5b9 fractions (the membrane attack complex), and platelets are activated. Other factors that may be responsible for venous thrombosis in Virchow’s triad are obesity, fluid and electrolyte imbalances, immobility, and oncological diseases, which, when combined with SARS-CoV-2 infection, may have adverse effects on the patient [23]. Due to this hypercoagulability, patients with COVID-19 are at increased risk of myocardial infarction, ischemic lesions, and venous thromboembolism, which can put pressure on the cardiac system and lead to fatal cardiovascular complications.

Another way vascular damage occurs is when SARS-CoV-2 infection disrupts mitochondrial function, leading to increased production of mitochondrial reactive oxygen species (mtROS). This excess mtROS causes cellular damage and accelerates endothelial cell aging [24]. It also activates TGF-β, promoting endothelial-to-mesenchymal transition (EndMT), which contributes to fibrosis and plaque instability. The virus can also directly impair mitochondrial regulation by forming protective double-membrane vesicles (DMVs), reducing viral clearance and sustaining infection. Additionally, mtROS triggers the release of pro-inflammatory cytokines, contributing to the cytokine storm observed in severe COVID-19 cases [24]. While the mtROS hypothesis is thoroughly argued and presents a compelling theoretical framework, it is important to highlight that clinical validation is currently lacking. Further clinical studies are needed to substantiate these findings in real-world settings.

A study on mice published in *Proceedings of the National Academy of Sciences* (PNAS) in 2024 demonstrated that both the genetic expression of mitochondrially targeted catalase (mCAT) and pharmacological treatment with the antioxidant EUK8 in SARS-CoV-2-infected mice had the following effects: reduced weight loss and clinical severity of infection, lowered circulating mitochondrial DNA levels, decreased lung levels of HIF-1α, viral proteins, and inflammatory cytokines, and downregulated genes associated with glycolysis and innate immune responses [25]. The authors propose that mtROS inhibitors like EUK8 could offer a host-directed therapeutic strategy for COVID-19 and potentially post-COVID-19 conditions, given their role in modulating mitochondrial function and inflammatory responses. While the study provides compelling evidence in a murine model, clinical validation in human subjects is necessary.

Some COVID-19 patients develop long-term cardiovascular problems marked by ongoing endothelial dysfunction, inflammation, and immune dysregulation. Biomarkers like D-dimer and IL-6 remain elevated even a year post-infection. A study published by Wu, Xiaoming et al. suggests that persistent viral proteins may linger in tissues like the intestines or liver, contributing to symptoms and that vaccination may reduce these effects. At the same time, elevated cytokines and immune cell activity are linked to vascular injury, with lasting microcirculation damage seen via imaging investigations [26].

The SARS-CoV-2 virus directly affects the cardiac muscle cell as a consequence of binding to the angiotensin-converting enzyme 2 receptor, which is highly expressed in the cardiac tissue, which, by binding, reduces the cardioprotective effects of this enzyme and can lead to acute cardiac injury [27]. Cardiac injury, in combination with systemic inflammation and, respectively, local inflammation with the release of proinflammatory cytokines, leads to the inflammation of cardiomyocytes, or what is called viral myocarditis [28]. Due to this inflammatory state, we can also talk about the impairment of myeloid function with the production of autoantibodies against nuclear antigens, phospholipids, T- and B-cell antigens, chemokines and cytokines, the downregulation of human leukocyte antigen class II (HLAII), and the release of immature neutrophils into the circulation. All these pathophysiological processes contribute to the pathogenicity of the SARS-CoV-2 virus in the occurrence of myocarditis [28].

A study from an Indian hospital showed that 12.9% of hospitalized COVID-19 patients had elevated cardiac troponin, indicating cardiac injury. These patients were older (*p* = 0.002), had more severe illness (SpO_2_ < 90%, *p* < 0.001), lower oxygenation (*p* = 0.001), and worse PaO_2_/FiO_2_ ratios (*p* < 0.0001). Elevated troponin was associated with higher ICU admission (*p* < 0.0001), ventilation use (*p* < 0.0001), and mortality (*p* < 0.0001) [29].

The important inflammatory state in COVID-19 disease leads to the infiltration of atherosclerotic plaques by cytokines and proinflammatory cells, which implies their instability, culminating in plaque rupture, coronary thrombosis, and acute coronary syndrome [30]. The coronary artery vasa vasorum, located in the epicardial tissue, plays a key role in the formation of early atherosclerotic lesions. This area, rich in epicardial adipose tissue, contributes to immune surveillance and vascular homeostasis. In pathological states, like in SARS-CoV-2 infection, this adipose tissue becomes pro-atherogenic and pro-arrhythmogenic due to macrophage activation [31]. Patients with COVID-19 are at increased risk of acute myocardial infarction in the first 30 days from the onset of infection, regardless of the severity of the respiratory disease, according to a study published in 2024 [32].

A study published in 2025 on autoantibody development suggests that COVID-19 infection is linked to an increased production of autoantibodies, particularly in severe cases. These autoantibodies, including those targeting GAD65, are associated with conditions like Type 1 diabetes (T1D) and rheumatic diseases, and their presence correlates with COVID-19 severity. Additionally, antibodies related to thrombotic conditions, like anti-cardiolipin, suggest a pro-thrombotic state in COVID-19 patients. The study highlights a need for autoimmune monitoring in COVID-19 patients to better understand the long-term implications and the development of autoimmune diseases following infection [33].

## 5. Pre-Existing Cardiovascular Disease

Several studies published since the onset of the COVID-19 pandemic have highlighted this association, emphasizing the vulnerability of this population to both acute and long-term complications like the one by Karakasis et al. that provided a comprehensive review of vascular alterations following COVID-19 infection, reinforcing the notion that individuals with cardiovascular comorbidities face a heightened risk of severe disease progression and poorer prognosis due to the compounded effects of viral-mediated endothelial damage and systemic inflammation [24]. Thus, a documented history of cardiovascular disease has been recognized as a significant risk factor for adverse outcomes in COVID-19 patients since the early stages of the pandemic. This association was quickly identified in clinical observations and epidemiological studies, raising widespread concern about the need for vigilant monitoring and specialized care in this vulnerable population. Rus et al. (2024), in their systematic review of acute myocardial infarction during the COVID-19 pandemic, emphasized that patients with pre-existing cardiovascular conditions not only faced a greater risk of severe disease but also demonstrated poorer long-term outcomes, underscoring the critical need for integrated cardiovascular care in the context of COVID-19 [34].

A study conducted in the province of Nova Scotia in Canada on a population of over 900,000 people demonstrated that due to the reduction in the provision of medical services, which were reduced globally in favor of medical care provided to patients with COVID-19, mortality and morbidity rates increased [35]. This study also highlights the impact that home isolation and restricted access to healthcare have had on the number of deaths and cardiovascular complications.

### 5.1. Atherosclerosis

The most important cause of cardiovascular disease is atherosclerosis, a fact recognized by the specialized literature and demonstrated over the years [36]. Atherosclerosis, as a disease of the vascular endothelium, is directly influenced by the tropism of the SARS-CoV-2 virus on the vascular endothelium, a relationship that is also influenced in the opposite direction [37].

Given the predominantly older age of patients hospitalized with SARS-CoV-2 infection and the direct correlation between age and more advanced atherosclerosis, through the direct effects of the virus on the vascular endothelium, a study published in 2023 by Makarova et al. shows a higher prevalence of acute coronary syndromes and thrombotic phenomena correlated with moderate and severe forms of COVID-19 disease [38]. Another study conducted on a population of patients identified positive for SARS-CoV-2 virus testing in the United States showed that an increased atherosclerotic disease risk score, predictive for a period of ten years, was correlated with worse outcomes and more severe forms of the disease [39].

### 5.2. Hypertension

The theory proposing a close link between the SARS-CoV-2 virus and hypertension is based on the shared affinity for the angiotensin II-converting enzyme receptor. The study conducted by Gallo et al. since the beginning of the pandemic shows a prevalence of pre-existing hypertension of over 50% [40] in patients hospitalized with moderate and severe forms of the disease [41]. Furthermore, hypertension was among the most common comorbidities associated with patients requiring intensive care due to infection with the SARS-CoV-2 virus [42].

The development of hypertension is closely linked to aging, with an incidence of over 60% in the elderly, thus being associated with more severe forms of COVID-19 disease and related complications [43]. The World Health Organization states that poor control of hypertension doubles the risks of a severe form of COVID-19, placing hypertension among the most important prognostic factors [1].

Hypertension, due to inflammation that directly affects the respiratory tree and an inadequate immune response, delays the elimination of the SARS-CoV-2 virus from the body [44]. Thus, the presence of this comorbidity is associated with a more severe form of COVID-19 by delaying viral clearance and decreasing the elimination of the virus from the body [45].

### 5.3. Diabetes

While a precise number of COVID-19 cases among diabetic patients is not readily available, substantial evidence from the Centers for Disease Control and Prevention (CDC) and the World Health Organization (WHO) underscores the heightened risk and adverse outcomes faced by individuals with diabetes when infected with the SARS-CoV-2 virus [1,3]. The CDC identifies diabetes as a significant risk factor for severe COVID-19 illness and states that individuals with diabetes are more likely to experience complications such as hospitalization, intensive care unit admission, mechanical ventilation, and death compared to those without diabetes [3]. Supporting this, a WHO analysis focusing on 13 African countries revealed that the case fatality rate for COVID-19 patients with diabetes was 10.2%, substantially higher than the 2.5% observed in the general COVID-19 patient population [1].

Patients diagnosed with diabetes, whether type 1 or 2, are more susceptible to infection with the SARS-CoV-2 virus through several mechanisms: increased expression of angiotensin-converting enzyme 2 receptors that increases susceptibility to infections [46]; reduced activity of Natural Killer cells that is associated with poor glycemic control [47]; decreased activation and phagocytic function of macrophages [48]; and by facilitating viral replication in the context of inflammatory syndrome [49].

Since the early part of the pandemic, several studies have shown an increase in mortality in people with diabetes and the association of the severity of COVID-19 disease with poor glycemic management in large-scale studies conducted on English [50] and Chinese populations [51]. At the same time, a significant correlation has been observed between mortality caused by COVID-19 and the proportion of obese adults in over 140 countries, with a majority of the obese population concentrated in high-income countries [52].

Recently, a link between COVID-19 and the de novo occurrence of diabetes mellitus has been suggested [53], thus placing SARS-CoV-2 infection among the risk factors for the occurrence of this metabolic dysfunction. This risk increases with the severity of the infection but decreases with the passage of time since recovery [54].

### 5.4. Atrial Fibrillation

Atrial fibrillation is one of the most common rhythm disorders, which does not delay appearing in the pathology of SARS-CoV-2 infection as a common pathology among hospitalized patients with moderate and severe forms of the infection. Both pre-existing and new-onset atrial fibrillation are associated with an increased risk of mortality, according to a meta-analysis by Li et al. [55]. Another study found an 11% incidence of this rhythm disorder among hospitalized COVID-19 patients and a direct association with short-term mortality [56]. At the same time, atrial fibrillation present before SARS-CoV-2 infection is associated with an increased risk of ARDS and, therefore, a poorer prognosis [57].

## 6. Acute Complications

### 6.1. Acute Myocardial Injury

Elevated cardiac troponin levels, without imaging or electrocardiographic evidence of ischemia, define what is called acute myocardial injury and represent one of the most common and early cardiac abnormalities in the acute phase of SARS-CoV-2 infection [58]. Both early studies [59] and more recent studies [60] have found that elevated cardiac troponin levels are correlated with disease severity, increased mortality, and the development of arrhythmias. Acute myocardial infarction is an acute myocardial injury with clinical evidence of ischemia and is the most common cardiovascular injury in patients with COVID-19 [61]. It is worth noting that people with pre-existing cardiovascular disease are more susceptible to myocardial injury compared to those without.

A Swedish study of 80,000 patients with confirmed SARS-CoV-2 infection showed an increased incidence of acute myocardial infarction in the first four weeks after infection [62]. A 2021 systematic review showed that among patients with acute myocardial injury, over 40% required intensive care and mechanical ventilation, and over 50% of them developed ARDS [63]. Evidence of cardiac ischemia may result in a three-fold increased risk of mortality, according to Lala, Anuradha, et al. [64].

These results contrast with epidemiological data from the beginning of the pandemic, when, due to the avoidance of hospital environments and incomplete diagnosis of critically ill patients, the rate of hospitalizations for acute cardiac events experienced a significant decline. Two meta-analyses published in 2021, which include over 30 studies related to acute cardiac events, show that cardiac lesions are the most common cardiac sequelae of this infection, with the myocardium being the part of the heart most frequently involved, followed by cardiac arrhythmias [65,66].

### 6.2. Cardiac Arrhythmias

A comprehensive review published in *Current Cardiology Reports* identified multiple arrhythmic phenomena associated with COVID-19 infection, ranging from relatively benign conditions such as transient sinus bradycardia to life-threatening conditions such as ventricular tachyarrhythmias and sudden cardiac death, explained by the association with systemic inflammation, hypoxemia, direct myocardial damage, and electrolyte imbalances. A variety of arrhythmic manifestations have been described in these patients, including atrial fibrillation, atrial flutter, sinus node dysfunction, atrioventricular blocks, and ventricular tachyarrhythmias, but atrial fibrillation is the most common and ranks first as the arrhythmia observed in COVID-19 patients. At the same time, the development of arrhythmias in patients hospitalized with COVID-19 has been associated with a higher risk of in-hospital death [67]. Arrhythmias in patients admitted to intensive care units seem to comprise the majority of cases of arrhythmias induced by the SARS-CoV-2 virus. This is underlined by the significant decorrelation of intensive care status at admission, with a 10-fold increase in the chances of developing atrial fibrillation, ventricular tachycardia, and bradyarrhythmia [68].

In an international study of 4526 patients from 12 countries, of whom 827 were identified with arrhythmia, the most common rhythm disorders developed were atrial arrhythmias (81.8%), followed by ventricular arrhythmias (20.7%) and bradyarrhythmias (22.6%). Regional particularities were also mentioned in this study, which showed a lower incidence of atrial fibrillation in Asia compared to other continents. At the same time, the majority of patients who developed cardiac arrhythmias required intensive care unit care and mechanical ventilation, with only about half of these patients (51%) surviving to hospital discharge. Thus, cardiac arrhythmias have been shown to be common and associated with high morbidity and mortality among hospitalized patients with COVID-19 infection [69].

### 6.3. Acute Myocarditis

Although differentiating acute myocardial injury from myocardial inflammation poses a challenge to clinicians in making the correct diagnosis, as myocarditis is characterized by elevated cardiac troponin levels without evidence of cardiac ischemia, SARS-CoV-2 infection has been associated with an increased incidence of myocarditis and may lead to severe complications [70]. Several studies have investigated this association and have provided valuable insights into the risks and mechanisms involved [24,33,34].

A study published in the *Journal of the American Medical Association* analyzed the risk of myocarditis after vaccination with messenger RNA vaccines against COVID-19. The results showed that although there is an increased risk of myocarditis after vaccination, this is significantly lower compared to the risk associated with SARS-CoV-2 infection. The study concluded that the benefits of vaccination outweigh the potential risks of myocarditis. The results, based on data from over 1 billion vaccinated individuals, showed just over 1000 cases that fit the definition of myocarditis, with a median age of 21 years and a median time to onset of symptoms of 2 days, and most of them were male [71].

A recent large-scale study published in *Vaccines* in 2024 clarified the relative risks of myocarditis following mRNA vaccination compared to SARS-CoV-2 infection [72]. According to this 2024 analysis, the incidence of myocarditis following mRNA COVID-19 vaccination was estimated at 1 to 10 cases per 100,000 vaccine recipients, with the highest rates observed in males aged 16–29. In contrast, SARS-CoV-2 infection was associated with a markedly higher myocarditis risk of approximately 150 cases per 100,000 infections [72]. When directly compared, the risk of developing myocarditis from SARS-CoV-2 infection was approximately 11 to 150 times greater than after mRNA vaccination, depending on age and sex [72]. These findings reinforce the favorable risk–benefit profile of mRNA vaccines, especially considering their role in preventing severe COVID-19 outcomes, including myocarditis itself.

The EMA (European Medicines Agency) has declared COVID-19 vaccination safe and is continuously monitoring the safety of COVID-19 vaccines to ensure that any potential risks are detected and managed as soon as possible. Most known side effects are mild and short-lived. Serious adverse reactions can occur but are very rare. Fatal outcomes have been reported in a very small number of these rare cases [73]. The CDC (Centers for Disease Control and Prevention in the United States) has declared the vaccines against the SARS-CoV-2 virus safe, whether they are mRNA or protein subunit vaccines. Currently, in the US, it is recommended that everyone aged 6 months and older be vaccinated with the updated COVID-19 vaccine (for the 2024–2025 season), including women who are pregnant, breastfeeding, or trying to conceive, thus once again underlining their safety [3].

### 6.4. Thromboembolism

A review by Othman, Hanies Yuhana, et al., published in 2024 [74], studied the incidence of venous thromboembolic events in patients with COVID-19 and found that it varied depending on the facilities in the hospital environment and the diagnostic protocols used by medical personnel. Despite prophylaxis administered to these patients since the beginning of the pandemic, the risk of thromboembolism was significant in patients hospitalized with moderate and severe forms of the disease, and in intensive care units, the risk was even higher. At the same time, this analysis also concludes a higher mortality rate in patients infected with the SARS-CoV-2 virus and who develop thromboembolic complications [74].

As for patients with mild forms of the disease, treated on an outpatient basis or at home, Xie J et al. describe in their study, published in 2022, the existence of genetic and clinical risk factors associated with an increased risk of patients developing thromboembolic phenomena. The effectiveness and importance of vaccine prophylaxis are also emphasized, the risk being much lower in people with complete vaccination schemes against this infection [75].

Similarly, there is also a study from the beginning of the pandemic that emphasizes the favorable effects of thromboprophylaxis in hospitalized patients with moderate or severe forms of infection, with increased levels of D-dimers and C-reactive protein during hospitalization. At the same time, the importance of the selective triage of these patients is highlighted, given the low risk of thromboembolic phenomena 6 weeks after discharge of patients whose inflammatory marker values were not increased by the type of hospitalization [76].

## 7. Long-Term Cardiovascular Effects

According to the World Health Organization, the term “post-COVID-19” is defined as a set of symptoms such as chronic fatigue, shortness of breath, cognitive dysfunction, and other symptoms with an impact on daily life, which appear in people with a possible or confirmed history of infection with the SARS-CoV-2 virus approximately 3 months after the initial infection, lasting at least 2 months and which cannot be explained by another diagnosis [1]. The CDC (Centers for Disease Control and Prevention in the United States of America) states about the post-COVID-19 condition that there are patients who have been infected with the SARS-CoV-2 virus, in whom symptoms persist for weeks or months after the acute episode, regardless of the initial form of the infection (severe or mild) [3].

A study published by A.B. Sherestha et al., including over eight million participants from seven studies, found that post-COVID-19 patients had significantly higher odds of cardiovascular outcomes (OR > 1, *p* < 0.05) compared to controls [77]. This meta-analysis provides valuable quantitative data on the prevalence of cardiovascular complications in individuals experiencing post-COVID-19, as summarized in Table 1, underscoring the significant burden of cardiovascular symptoms among post-COVID-19 patients.

A retrospective double-cohort study conducted in the United States of America associates an increased risk of cardiovascular events approximately 10 months after infection with the SARS-CoV-2 virus, regardless of the presence or absence of symptoms associated with this respiratory infection, a risk supported by pathophysiological and clinical phenomena [78]. Long-term cardiac manifestations, the so-called “long-COVID”, which is the same as the “post COVID-19 condition”, include persistent arrhythmias, inflammatory conditions, ischemic disease, heart failure, and dysautonomia [79]. Current approaches to the management of this pathology emphasize symptom monitoring based on medical history, vital signs, and clinical examination, along with specific diagnostic investigations, starting with the electrocardiogram. Active individuals, such as athletes or those involved in strenuous activities, may benefit from troponin testing and an echocardiogram as part of the initial evaluation. Treatment of these symptoms is based on progressive physical exercise and supportive measures, and in the event of the appearance or worsening of a cardiovascular condition, it should be managed according to the latest clinical guidelines.

Defined by the presence of symptoms associated with SARS-CoV-2 infection more than 4 weeks after onset, the post-COVID-19 condition is increasingly prevalent among patients who have experienced this disease. Symptoms reported by survivors, ranging from mild to severe in the cardiovascular sphere, are concluded by Ramadan et al. in a retrospective review of several studies as dyspnea, angina pectoris, palpitations, and dizziness [80]. Symptoms related to the post-COVID-19 condition are diverse and prolonged, affecting both physical and psychological health, as described by Chiyoung et al. in a systematic review [81]. The most common physical symptoms include dyspnea, particularly in hospitalized patients, and fatigue, which shares similarities with chronic fatigue syndrome. Psychological symptoms such as anxiety, depression, memory problems, and sleep disorders are also frequently reported [81]. These symptoms can result from both the biological impact of the virus and the stress associated with the pandemic.

The following are mentioned as risk factors for the post-COVID-19 condition: associated comorbidities, older age, the severity of the initial disease, patients presenting with chronic fatigue 10 weeks after infection with the SARS-CoV-2 virus, patients with a history of neuropsychiatric pathologies, and female gender due to more important hormonal changes and a more pronounced immunological response [82]. Also, COVID-19 survivors who required care in the Intensive Care Unit and who were mechanically ventilated (invasively or non-invasively) were much more likely to develop long-COVID syndrome approximately 3 months after the initial infection [83].

At the same time, associations were also made between biomarkers associated with the severity of SARS-CoV-2 infection, such as D-dimers, nitrogen retention, increased levels of C-reactive protein or IL-6, which were correlated with pulmonary dysfunction, and the occurrence of the post-COVID-19 condition [84]. Low lymphocyte counts during the acute episode have been correlated with an increased risk of tachycardia in patients with the post-COVID-19 condition [85], and elevated cardiac troponin levels have been correlated with fatigue in patients with the post-COVID-19 condition [86].

Cardiac arrhythmias were observed both in the acute phase of infection and as part of the post-COVID-19 condition, being one of the most common cardiac symptoms during acute infection, and most patients had no prior history of cardiac arrhythmias, as shown in a retrospective analysis of patients hospitalized with COVID-19 infection worldwide on 4526 patients [69]. In this study, of those who developed arrhythmias, 81.8% had atrial arrhythmias, 20.7% had ventricular arrhythmias, and 22.6% had bradyarrhythmias [69]. In another study on 204 patients, Ingul and colleagues performed 24 h ECG monitoring in patients 3–4 months after COVID-19, detecting arrhythmias in 27% of them, with ventricular extrasystoles being the most common [87].

**Figure 2:**
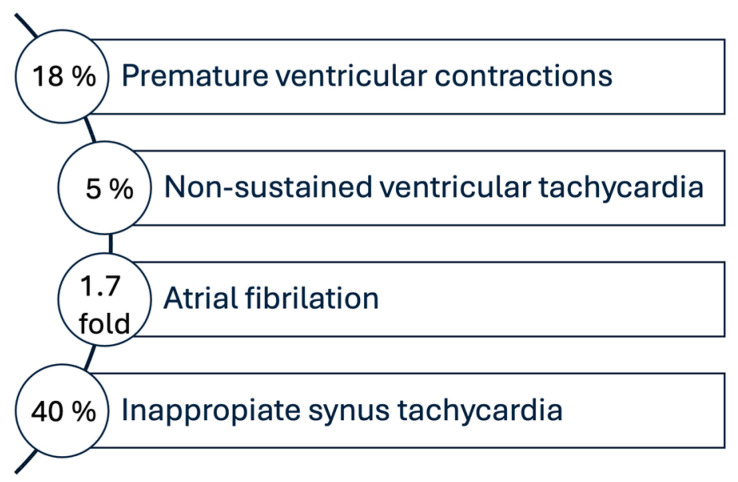
Post-COVID-19 cardiac arrhythmias: prevalence and patterns. Data obtained from the study by Zuin M. et al., 2024 [88].

More recent studies, like the meta-analysis published by Zuin, Marco, et al. in 2024 [88], have demonstrated that cardiac arrhythmias are a prevalent complication in individuals experiencing the post-COVID-19 condition, with an overall incidence estimated between 10% and 20%, particularly affecting those with severe acute infection. Among the most common arrhythmias observed, premature ventricular contractions were reported in 18% of post-COVID-19 patients, with an average burden of approximately 1300 premature ventricular contractions per day (*p* < 0.01), suggesting ongoing myocardial irritability. Additionally, non-sustained ventricular tachycardia was identified in 5% of patients, primarily in those with underlying structural heart disease (OR: 2.3; 95% CI: 1.4–4.0; *p* = 0.008). Atrial fibrillation also appeared more frequently, with a 1.7-fold increased incidence compared to matched control populations (HR: 1.7; 95% CI: 1.2–2.3; *p* = 0.002), emphasizing a significant elevation in long-term arrhythmic risk. Moreover, inappropriate sinus tachycardia was observed in up to 40% of post-COVID-19 patients, especially among young women without prior cardiovascular conditions (*p* < 0.001). These findings, seen in Figure 2, underscore the importance of ongoing surveillance and cardiac monitoring in the post-acute phase of COVID-19 recovery [88].

Autonomic dysfunction is considered the main cause of neurocardiogenic abnormalities, affecting blood flow, especially in the brain and structures above the heart. This can lead to orthostatic disorders, such as postural orthostatic tachycardia syndrome (POTS) or orthostatic hypotension, characterized by chronic orthostatic intolerance, manifested by an increase in heart rate of at least 30 beats per minute within 10 min after standing, without the development of significant hypotension, and can also lead to episodes of syncope. In addition, clinical manifestations are often associated with symptoms such as fatigue, exercise intolerance, dizziness, difficulty concentrating, memory problems, and sleep disturbances [89]. Depression, anxiety, and frequent headaches, including migraines, are also common. The mental fog that occurs in the post-COVID-19 condition may be caused by factors such as cardiovascular deconditioning, post-traumatic stress, or dysautonomia. The relationship between the post-COVID-19 condition and cognitive impairment could be explained by autonomic dysfunction, specifically by reduced sympathetic nervous system activity (alpha-adrenergic dysfunction), which contributes to the occurrence of orthostatic disorders [89].

**Figure 3:**
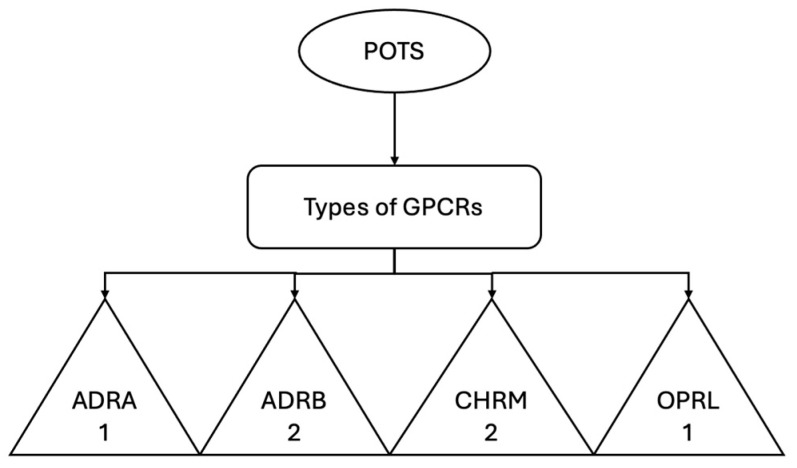
Post-COVID-19 postural orthostatic tachycardia syndrome (POTS) and serum G-protein-coupled receptor (GPCR). Data obtained from the study by Johansson, M. et al., 2024 [90].

A recent study published in *Hypertension* examined the relationship between serum G-protein-coupled receptor (GPCR) activity and the severity of orthostatic symptoms (see Figure 3) in patients diagnosed with postural orthostatic tachycardia syndrome (POTS) [90]. The study, which included 48 patients with POTS and 25 healthy controls, showed that patients with POTS had significantly higher scores on the Orthostatic Hypotension Questionnaire, indicating more severe orthostatic symptoms compared with controls (*p* < 0.001). Serum activity against several GPCRs—including α1-adrenergic receptors (ADRA1), β2-adrenergic receptors (ADRB2), muscarinic M2 receptors (CHRM2), and opioid receptors OPRL1—was significantly higher in patients with POTS compared with controls, with *p*-values ranging from <0.05 to <0.001. Linear regression analyses revealed a positive correlation between increased activity against ADRB2 and the severity of orthostatic symptoms, as measured by questionnaire scores (β = 0.45, 95% CI: 0.20–0.70, *p* = 0.001) [90]. These results support the hypothesis of an autoimmune component in the pathogenesis of POTS and suggest that serum GPCR activity may be a relevant biomarker for symptom severity and diagnosis.

The post-COVID-19 condition poses significant cardiovascular risks in African populations, characterized by a unique interplay of epidemiological, clinical, and socioeconomic factors [91]. Cardiovascular sequelae, such as myocarditis, arrhythmias, and thromboembolic events, are increasingly reported but remain underdiagnosed due to limited access to advanced diagnostics (e.g., cardiac MRI) and healthcare disparities. High rates of pre-existing comorbidities, including hypertension, diabetes, and HIV, prevalent across Africa, amplify susceptibility to post-COVID-19 cardiac injury. Persistent symptoms like chest pain, palpitations, and exertional dyspnea are common, alongside severe outcomes such as myocardial fibrosis and elevated troponin levels indicative of subclinical damage [91].

In South Asian populations, long-term post-COVID-19 cardiovascular manifestations are shaped by a confluence of biological, socioeconomic, and environmental factors, as highlighted in the study “Post COVID-19 Cardiovascular Complications in South Asia: A Regional Perspective” [92]. South Asia, with its high prevalence of metabolic comorbidities (e.g., diabetes and hypertension) and genetic predispositions, faces a heightened burden of post-COVID-19 cardiac sequelae. Common cardiovascular pathologies include myocarditis, arrhythmias, ischemic heart disease, and thromboembolic events, often exacerbated by pre-existing conditions and delayed healthcare access. Persistent symptoms such as chest pain, palpitations, and exertional dyspnea are frequently reported. Severe outcomes include myocardial fibrosis, elevated cardiac biomarkers (troponin and NT-proBNP), and microvascular dysfunction. South Asian patients also show a higher incidence of post-COVID-19 thromboembolic complications (e.g., pulmonary embolism and deep vein thrombosis) linked to chronic inflammation and hypercoagulability. Autonomic dysfunction, including postural orthostatic tachycardia syndrome (POTS), is increasingly recognized, likely due to viral-induced neuroinflammation. Cultural stigma around chronic symptoms and the underprioritization of post-acute care further hinder timely intervention. Resource constraints, sparse rehabilitation programs, and insufficient research into region-specific genetic and epigenetic drivers exacerbate disparities, leaving long-term cardiovascular sequelae underaddressed [92].

A study of 133 patients infected with the SARS-CoV-2 virus, which followed their evolution 3 months after the infectious episode, illustrated that flow-mediated dilation was reduced in patients who had been infected with the disease compared with a group without a history of infection [93]. Thus, this infection was associated with endothelial dysfunction. The association between endothelial dysfunction and cardiovascular complications resulting from the infection leads to an increased risk of developing hypertension, a fact demonstrated in a study of 153 post-COVID-19 patients, in which the majority presented higher systolic and diastolic blood pressure values than before the infection [94].

The diagnosis of the post-COVID-19 condition can be managed in a variety of healthcare settings. A comprehensive clinical history is crucial for diagnosing the post-COVID-19 condition, as there is no definitive biomarker [80]. The post-COVID-19 condition symptoms often overlap with other conditions, leading to frequent misdiagnoses [95]. Echocardiography, ECG, cardiac MRI, and cardiopulmonary exercise testing are methods that can help identify patients with the post-COVID-19 condition [96].

Various echocardiographic abnormalities have also been found in the hearts of patients with post-COVID-19 syndrome. A study published in 2025 by Samy et al. identified a significant correlation (*p*-value > 0.001) between the post-COVID-19 condition and symptoms like shortness of breath or chest discomfort, alongside changes in 3D echocardiographic strain patterns. The mean left ventricular global longitudinal strain was significantly lower in the post-COVID-19 group (−16.06 ± 4.36) compared to the control group (−17.9 ± 2.57) [97]. This decrease in left ventricular global longitudinal strain is indicative of subclinical myocardial dysfunction and was found in over 85% of patients with the post-COVID-19 condition. However, more commonly used markers of left ventricular function, such as ejection fraction and wall motion abnormalities, were less frequently observed in this study [97].

Other problems found in echocardiographic imaging include left ventricular pump dysfunction, as well as diastolic dysfunction, global myocardial hypokinesia, and pulmonary hypertension. A study published in 2025 on the south-eastern population of Romania reveals a high frequency of newly developed left ventricular (LV) dysfunction in the third year post-COVID-19 hospitalization in patients with no prior cardiac history [98]. No correlation was found (*p* > 0,001) between cardiac changes and the severity of the infectious episode, suggesting that cardiac damage may not be directly linked to disease severity but rather to persistent viral effects on myocardial tissues and other factors like aging, lifestyle, or other viral infections, that may contribute to left ventricular dysfunction [98].

A study published in the *Journal of Interventional Cardiac Electrophysiology* explores changes in heart rate variability (HRV) and autonomic function in individuals with a history of COVID-19 by HRV analysis, which measures the function of the autonomic nervous system. When comparing post-COVID-19 patients to healthy ones, time domain indices of HRV analysis and root mean square of successive RR interval differences were significantly higher in post-COVID-19 patients (*p*  <  0.05 for all) [99].

Cardiopulmonary exercise testing (CPET) should be prioritized for patients with persistent exertional intolerance (>3 months post-infection), using predefined thresholds for abnormality. A ventilatory efficiency (VE/VCO2) slope ≥34, peak oxygen uptake (VO2) <85% predicted, or heart rate recovery ≤12 beats/minute after 1 min of rest constitute criteria for further cardiac evaluation [100]. CPET is performed using a cycle ergometer and continuous analysis of respiratory gas exchange breath by breath. The system must be calibrated before each test, and after a 2 min rest period before exercise, the test begins at an initial workload of 20 to 40 W and increases by 10 to 25 W each minute until the test subject reaches exhaustion, usually between 8 and 12 min. Oxygen saturation and blood pressure are measured, and the ECG is recorded during the test [101].

While cardiopulmonary exercise testing (CPET) remains the gold standard for assessing cardiorespiratory fitness in post-COVID-19 syndrome, traditional maximal-effort protocols risk exacerbating post-exertional symptom exacerbation, severely impacting patients’ quality of life. Emerging evidence, including a meta-analysis by Sorensen et al., supports using submaximal endpoints (e.g., ventilatory threshold) rather than exhaustive testing to avoid symptom flares [102]. Protocols must prioritize patient safety by integrating insights from lived experience, adopting adaptive designs (e.g., 2-day CPET), and ensuring wrap-around support to mitigate risks. Research and clinical practice should be centered on patient-informed approaches to develop safe, meaningful assessments that advance restorative care without worsening outcomes.

Cardiac MRI emerged as a critical tool during the COVID-19 pandemic due to its non-invasive, comprehensive imaging capabilities, which minimize healthcare exposure and infection risk. By providing multicomponent assessments of cardiac function, ischemia, viability, and valvular integrity in a single session, cardiac MRI aids in differentiating post-COVID-19 cardiovascular conditions and optimizes medical care decisions. By combining cine imaging, contrast-enhanced angiography, and late gadolinium enhancement, this specific type of MRI effectively evaluates thrombi and structural anomalies without major risks [103]. Its ability to maintain diagnostic accuracy despite rhythm irregularities further solidifies its role in pandemic-era cardiovascular care, balancing efficiency, safety, and clinical utility. Cardiac MRI referrals should follow a stepwise approach: (1) persistent troponin elevation (>14 days) with concurrent ECG abnormalities (ST-T changes and arrhythmias), (2) echocardiographic evidence of reduced left ventricular ejection fraction (<50%) or regional wall motion abnormalities, or (3) ongoing chest pain/dyspnea despite normal initial testing [103].

Due to the cardiovascular burden of SARS-CoV-2 infection, current cardiovascular guidelines have been adapted so that the diagnostic evaluation of post-COVID-19 cardiac syndrome is currently based on a rigorous history and symptomatology. In patients with persistent and unexplained cardiopulmonary symptoms, cardiopulmonary exercise testing is indicated to identify and differentiate cardiac, pulmonary, and vascular problems or a combination of these. As for the management of other cardiac conditions related to the post-COVID-19 condition, the measures in the current guidelines suggest supportive measures, cardiopulmonary rehabilitation, and psychological counseling [104].

One of the most important questions would be how to prevent long-term complications of SARS-CoV-2 infection. Firstly, it has been shown that antiviral therapy, in the first days of acute COVID-19 infection, is significantly (27.5%; 95% CI, 25.3–59.1) preventing the post-COVID-19 condition, as stated in a meta-analysis from 2023 by Yu Jung Choi et al. [105]. One proposed explanation for the post-COVID-19 condition is that SARS-CoV-2 triggers persistent inflammation in the body. Based on this theory, initiating antiviral therapy early in the course of infection could help suppress viral replication, lower the overall viral burden, and potentially alleviate COVID-19-related symptoms.

Secondly, vaccination against COVID-19 has been shown to be effective against long-term complications, even after only one dose of vaccination (OR 0.539, 95% CI 0.295–0.987, *p* = 0.045, N = 257,817), as shown in a meta-analysis conducted by Felicia Ceban et al. [106]. Vaccination is theoretically expected to facilitate faster viral clearance, mitigate acute immune reactions and minimize tissue damage, and help stabilize immune function, potentially reducing the likelihood of secondary autoantibody production.

A thorough medical and symptom history and follow-up visits are essential to track a patient’s complex trajectory, helping them understand their symptoms and validate the diagnosis, a validation that can strengthen the physician–patient relationship. Clinical management, including a multidisciplinary rehabilitation approach, is recommended for patients experiencing significant symptoms within the first six months after initial infection with the SARS-CoV-2 virus. Rehabilitation may include pacing, physiotherapy, psychological support, cognitive therapy, and lifestyle adjustments.

## 8. Conclusions

**Figure 4:**
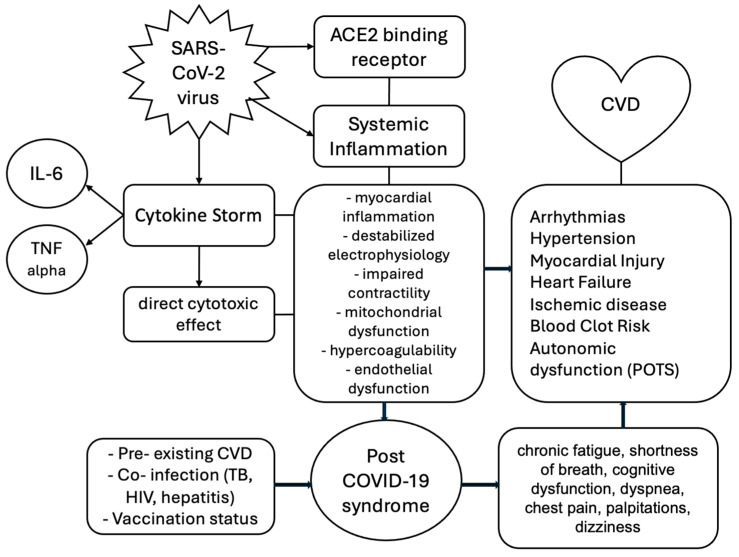
The interplay between SARS-CoV-2 virus and cardiovascular disease. CVD = cardiovascular disease; TB = tuberculosis. Viral entry via ACE2 directly damages cardiomyocytes, while immune-mediated inflammation exacerbates pre-existing atherosclerosis and myocardial dysfunction. The virus has a bidirectional relationship with cardiovascular disease, as the viral infection alone can cause cardiovascular damage, but through the same mechanisms, it can aggravate pre-existing cardiovascular diseases.

To conclude, there is a complex bidirectional relationship between SARS-CoV-2 infection and cardiovascular health, as illustrated in Figure 4. The central component, COVID-19 (caused by SARS-CoV-2), is shown to impact the cardiovascular system through several interconnected pathophysiological mechanisms, including direct viral invasion via ACE2 receptors, systemic inflammation, and cytokine storms by inducing a direct cytotoxic effect. These processes contribute to a spectrum of acute and chronic cardiovascular diseases and complications, such as arrhythmias, hypertension, myocardial injury, heart failure, ischemic disease, hypercoagulability, and autonomic dysfunction (POTS). On the other hand, the post-COVID-19 condition is represented as a downstream consequence, with a broad spectrum of symptoms that could be signs of cardiovascular disease as a part of this syndrome. The diagram further incorporates contributing factors such as pre-existing cardiovascular disease, co-infections like tuberculosis, HIV/AIDS, or hepatitis, and vaccination status, which increase the risk of long-term cardiovascular sequelae in post-COVID-19 syndrome.

COVID-19 disease, caused by the SARS-CoV-2 virus, has revealed itself not merely as a respiratory illness but as a systemic disease with profound and lasting implications for cardiovascular health. The emergence of the post-COVID-19 condition underscores the chronic nature of the SARS-CoV-2 virus’s impact, particularly through persistent cardiovascular symptoms such as tachycardia, chest pain, myocardial injury, and endothelial dysfunction, even in individuals who experienced mild or asymptomatic infections. The virus’s exploitation of ACE2 receptors, induction of cytokine storms, systemic inflammation, endothelial damage, immune-mediated injury, and mitochondrial dysfunction form a complex pathophysiological cascade that contributes to acute and long-term cardiovascular complications.

Patients with pre-existing cardiovascular conditions, such as hypertension, atherosclerosis, and diabetes or co-infections like HIV, tuberculosis, or hepatitis, are disproportionately affected, facing elevated risks of severe illness and lingering sequelae. This interplay between pre-existing heart disease and post-viral pathology intensifies the burden on individuals and healthcare systems alike. The persistence of immune dysregulation and vascular dysfunction long after the resolution of the acute infection further highlights the necessity for sustained vigilance and care.

We emphasize the critical importance of recognizing post-COVID-19 symptoms by both healthcare professionals and patients in order to ensure timely intervention and access to appropriate medical care. Public health campaigns are essential to destigmatize the post-COVID-19 condition, educate the public, and encourage early medical consultation. Heightened awareness can help reduce delays in diagnosis and prevent the progression of long-term complications. It is essential that standardized clinical protocols and guidelines for the management of the post-COVID-19 condition be developed, validated, and widely disseminated to support consistent and effective care across healthcare systems.

Furthermore, multidisciplinary rehabilitation programs tailored specifically for individuals suffering from the long-term sequelae of SARS-CoV-2 infection must be established. These programs should address the physical, psychological, and cardiovascular impairments often seen in post-COVID-19 condition patients, aiming to restore function, improve quality of life, and reduce the overall burden on healthcare systems. Public health initiatives and educational campaigns are also vital to destigmatize the post-COVID-19 condition, encourage early medical consultation, and foster a comprehensive, compassionate approach to recovery.

Promoting continued vaccination against COVID-19 remains a key strategy, even in the post-pandemic phase. Updated booster vaccinations can help reduce the incidence and severity of acute infections and, consequently, lower the risk of developing the post-COVID-19 condition. Public health efforts should focus on raising awareness about the protective role of vaccination, not only in preventing infection but also in mitigating long-term complications.

These insights call for an urgent and sustained multidisciplinary response, integrating cardiology, infectious diseases, immunology, and public health. Long-term research is essential to unravel the full spectrum of COVID-19’s cardiovascular consequences, develop targeted therapies, and establish comprehensive surveillance and prevention strategies, especially for high-risk populations. Recognizing the cardiovascular dimension of COVID-19 is crucial not only for improving outcomes in affected patients but also for preparing global health systems to better confront future pandemics.

## Figures and Tables

**Table 1:** This table summarizes the pooled incidence rates of various cardiovascular complications observed in patients with post-COVID-19 syndrome.

Cardiovascular Manifestation	Pooled Prevalence (%) *	95% Confidence Interval *	Number of Studies Included *
Chest pain	10.1	6.4–15.5	9
Palpitations	9.8	5.4–17.2	7
Dyspnea	28.8	21.5–37.5	10
Hypertension	20.3	13.3–29.7	6
Myocardial injury	13.5	8.3–21.2	4
Arrythmia	9.6	5.2–17.0	5
Heart failure	5.8	2.8–11.6	3

* Data adapted from the study by Shrestha, A.B. et al., 2023 [77].
